# Investigation of the Potential Use of Calcium Alginate Capsules for Self-Healing in Porous Asphalt Concrete

**DOI:** 10.3390/ma12010168

**Published:** 2019-01-07

**Authors:** Shi Xu, Xueyan Liu, Amir Tabaković, Erik Schlangen

**Affiliations:** 1 Civil Engineering and Geosciences, Delft University of Technology, Delft 2628CN, The Netherlands; x.liu@tudelft.nl (X.L.); amir.tabakovic@dit.ie (A.T.); Erik.Schlangen@tudelft.nl (E.S.); 2 Research Enterprise and Innovation, Dublin Institute of Technology, Dublin D07 EWV4, Ireland; 3 School of Civil Engineering, University College Dublin, Dublin D04 K3H4, Ireland

**Keywords:** self-healing, asphalt, rejuvenation, calcium alginate capsules

## Abstract

Improving the healing capacity of asphalt is proving to be an effective method to prolong the service life of an asphalt pavement. The calcium alginate capsules encapsulating rejuvenator have been developed and proved to provide successful localized crack healing in asphalt mastic. However, it is not known whether this self-healing asphalt system will improve healing capacity of a full asphalt mix. To this aim, this paper reports on study which investigate effect of the calcium alginate capsules onto self-healing capacity of the porous asphalt mix. X-ray computed tomography (XCT) was used to visualize the distribution of the capsules in porous asphalt. The effect of the capsules on fracture resistance of porous asphalt concrete was studied by semi-circular bending (SCB) tests. A semi-circular bending and healing programme was carried out to evaluate the healing effect of these capsules in porous asphalt concrete. Indirect Tensile Stiffness Modulus (ITSM) tests were employed in order to investigate the influence of the capsules on the stiffness of the porous asphalt concrete. The results indicate that incorporating calcium alginate capsules significantly improve the healing capacity of porous asphalt concrete without compromising its performance.

## 1. Introduction

The asphalt healing phenomenon was first reported by Bazin in 1967 [1], since then, it captured attention of road engineers and material scientists. Self-healing became a hotspot in the research field of bituminous materials [2]. The healing mechanism can be explained as interpenetration and bonding, which means the cracking interface will disappear as a function of time that the cracking surfaces are brought in contact, and this phenomenon can be accelerated with a temperature increase [3]. In the life cycle of an asphalt pavement, the self-healing capacity of asphalt plays an important role against the fatigue damages, which is considered by pavement engineers in predicting the performance of asphalt pavement [2]. However, the intrinsic healing capacity of asphalt pavement itself is not sufficient to provide an efficient healing and significant life extension. Aimed to prolong the service life of asphalt pavement with self-healing technologies, more researches focused on the development of extrinsic healing methods in asphalt.

The induction healing methods, including induction heating and microwave heating, have been intensively investigated in the past ten years [4]. The concept lies on the induction heating of the embedded conductive particles to improve the healing capacity of a pavement [5,6,7,8]. The research findings indicate that the induction healing method has significant healing effect on the micro cracks in asphalt pavement. However, this technology could not reduce the ageing of asphalt or even accelerate the ageing with high temperature, which might results in a stiffer asphalt binder which leads to increased cracking and pavement failure.

Embedded rejuvenator encapsulation method provides an alternative extrinsic solution for self-healing asphalt. The concept is to add encapsulated healing agent (rejuvenator) in the asphalt mix, allowing the release of encapsulated healing agent on demand (upon cracking) and healing the crack by softening the aged binder allowing it flow which in turn closes the crack and repairs the damage [9]. To this aim, several encapsulation methods are developed, including epoxy capsules [10], Melamine-formaldehyde (MMF) modified capsules [11], calcium alginate capsules [9] and alginate fibres [12]. Among them, the calcium alginate capsules have the advantages of simple production process, low cost, environmental friendly and ability to encapsulate higher amounts of rejuvenator, which shows great potential for the application in self-healing asphalt [9,13].

Porous asphalt (PA), the open graded asphalt mix with a void content above 20%, was first designed and applied in 1972 in Netherlands [14]. The advantages of a PA include noise reduction, comfortable driving and reducing of splash and providing good water draining preventing water accumulation on the surface of the pavement. As a result, PA was implemented quickly in the asphalt pavement design in Netherlands and worldwide [15,16,17]. However, the high void content structure of porous asphalt concrete is prone to clogging, stripping, and accelerated aging [18,19]. Raveling of porous asphalt concrete, which is a failure at the surface of the pavement occurring within the stone-to-stone contact regions and causes the loss of aggregate particles from the road surface, is the main defect of porous asphalt [19]. It is caused by an increase of stiffness, reduction of relaxation capacity and formation of micro-cracks in the binder due to aging [20]. If the micro-cracks can be healed in the early stage, the problem of raveling can be prevented or delayed, as such the service life of porous asphalt will be prolonged [21].

**Figure 1:**
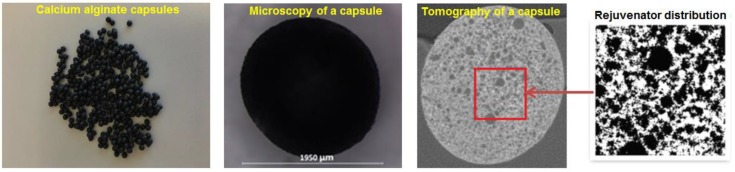
Calcium alginate capsules prepared by Xu et al. [9].

In a previous paper, Xu et al. [9] successfully prepared calcium alginate capsules Figure 1. The X-ray tomography image of the capsule indicates that the calcium alginate capsules have a special porous structure instead of a traditional core-shell structure, which small rejuvenator droplets are encapsulated by porous media within the shell. The capsules thermal stability and compressive resistance were tested through thermogravimetric analysis (TGA) and micro-compressive tests. Xu et al. [9] showed that the calcium alginate capsules are able to survive the asphalt production and compaction process. Furthermore, the healing effect of these capsules were investigated with a three-point-bending testing and healing programme on asphalt mastic beams. The results indicated that the healing capacity of asphalt mastic beams with calcium alginate capsules are significantly higher than reference beams.

Xu et al. [9] explored general potential use of calcium alginate capsules in self-healing asphalt by investigating the capsule properties and the healing effect in mastic. However, the efficiency of the calcium alginate capsule encapsulating rejuvenator healing system in a full asphalt mix is unknown. Tabaković et al. [22] reported that self-healing rejuvenation process using compartmented alginate fibre encapsulating rejuvenator has very small effect on improvement of fatigue properties of PA mix. However, alginate capsules have ability to deliver higher amount of healing agent to the damaged area and as such have a better ability to repair the damage and restore asphalt pavement physical and mechanical properties. Therefore, the objective of this study was to investigate how calcium alginate capsules encapsulating rejuvenator affect the mechanical properties and the healing capacity of porous asphalt concrete. To this aim, a porous asphalt mix with capsules was designed and compacted. A porous asphalt mixture without capsules was used as reference mixture. The distribution of the calcium alginate capsules was investigated by XCT. The healing capacity of the porous asphalt mix was evaluated by a testing and healing programme based on SCB tests. The standard ITSM test were employed to investigate how these capsules influence the stiffness of porous asphalt concrete. The fluorescence characteristics of the rejuvenator was used to track the transportation of rejuvenator on fractured surface.

## 2. Experimental Method

### 2.1. Materials

#### 2.1.1. Calcium-Alginate Capsules

Chemicals including sodium alginate, poly(ethylene-alt-maleic-anhydride) (PEMA) and CaCl_2_ were used to prepare these capsules. Since the calcium alginate capsules were manufactured following the same procedures as the previous research, as shown in Figure 1, the prepared capsules have a uniform diameter of 1.95 mm and a honey-comb like porous structure [9]. These capsules also possessed the same mechanical resistance and thermal stability as previous research [9]. The industrial rejuvenator R20 encapsulated in these capsules was provided by Latexfalt B.V., Koudekerk aan den Rijn, Netherlands. The other chemicals used in this research were purchased from Sigma Aldrich, St. Louis, MO, USA.

**Figure 2:**
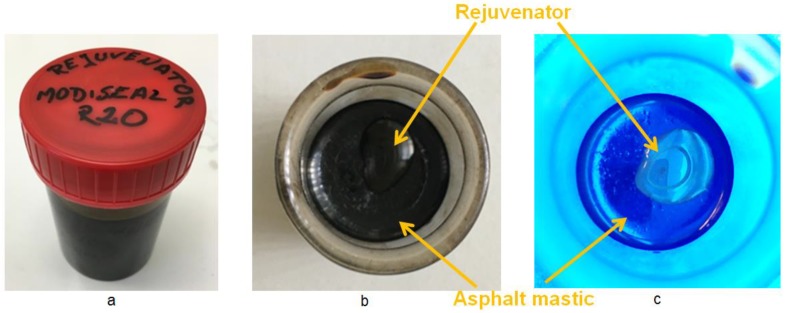
Rejuvenator R20: (**a**) normal appearance; (**b**) on asphalt mastic under visible light and (**c**) on asphalt mastic under UV light.

Figure 2 shows the rejuvenator used in this research. The rejuvenator named as R20 is black under normal visible light (Figure 2a), which makes it very hard to distinguish the rejuvenator from asphalt mastic (Figure 2b). However, the fluorescent component of the rejuvenator turns into brown color under ultraviolet (UV) light (Figure 2c), which allows the rejuvenator being tracked once released via the fracture of capsules.

#### 2.1.2. Porous Asphalt Mix and Test Specimens

The porous asphalt mix used in this study was based on the standard mix PA 0/11 (Table 1). Two types of mixtures were designed for the study, including a porous asphalt mix with capsules and a reference mix without capsules. According to the previous research, using calcium alginate capsules to replace 7% the volume of bitumen (8% by weight) possesses a higher healing efficiency. Thus, in this paper, the capsules were added in the porous asphalt mix by replacing 7% volume of the bitumen [9]. The bitumen used in the mix was the standard paving grade bitumen 70–100 provided by Vitol, London, UK. All the aggregates were Bestone (a kind of limestone widely used in PA construction in Netherlands, Bontrup, Amsterdam, Netherlands) or byproduct of Bestone. The porous asphalt test samples used in this study include cylinders for Indirect Tensile Stiffness Modulus (ITSM) tests and semi-circular samples for Semi Circular Bending (SCB) tests. All the samples were drilled from 50 cm × 50 cm × 5 cm asphalt slabs.

**Figure 3:**
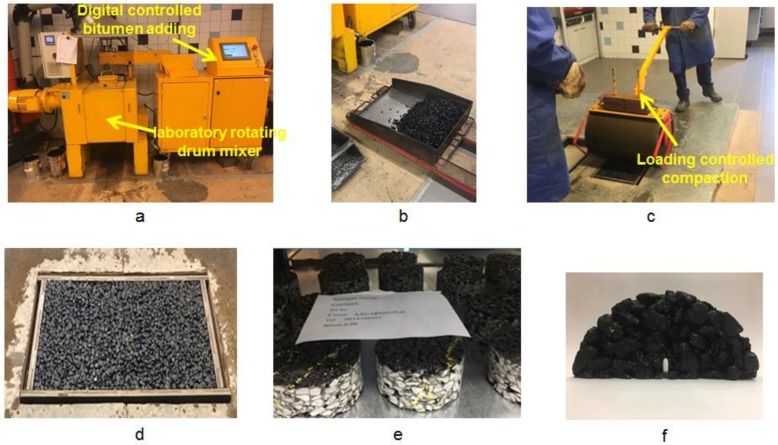
Porous asphalt slab preparation procedure: (**a**) mixing in laboratory rotating drum mixer; (**b**) weighing the mixture; (**c**) compaction with manually roller compactor; (**d**) prepared porous asphalt slab; (**e**) cylinder samples for ITSM tests and (**f**) semi-circular sample for SCB tests.

The asphalt slabs were prepared in Rosmalen Heijmans infra BV (Heijmans Infra, Rosmalen, Netherlands). To prepare a slab, the designed pre-heated materials were carefully mixed in the laboratory rotating drum mixer (Figure 3a). Then, the mixed materials were collected and weighed to meet the required amount of an asphalt slab (Figure 3b). Subsequently, the weighed asphalt mix was poured into a mold and compacted with a manual roller compactor (Figure 3c). After a series of compaction cycles, the porous asphalt slab was produced (Figure 3d).

For the ITSM tests, cylinder samples (Figure 3e) were drilled from the slabs and at least 6 cylinder samples were tested for each group. For the SCB tests, the semi-circular samples (Figure 3f) were acquired by sawing the asphalt cylinders in half and a notch was inserted in each SCB specimen and at least 6 SCB samples were tested for each group. For the XCT test, a small cylinder with 33.5 mm in diameter and 48.5 mm in height was drilled from mixture.

### 2.2. Testing Methods

#### 2.2.1. XCT

**Figure 4:**
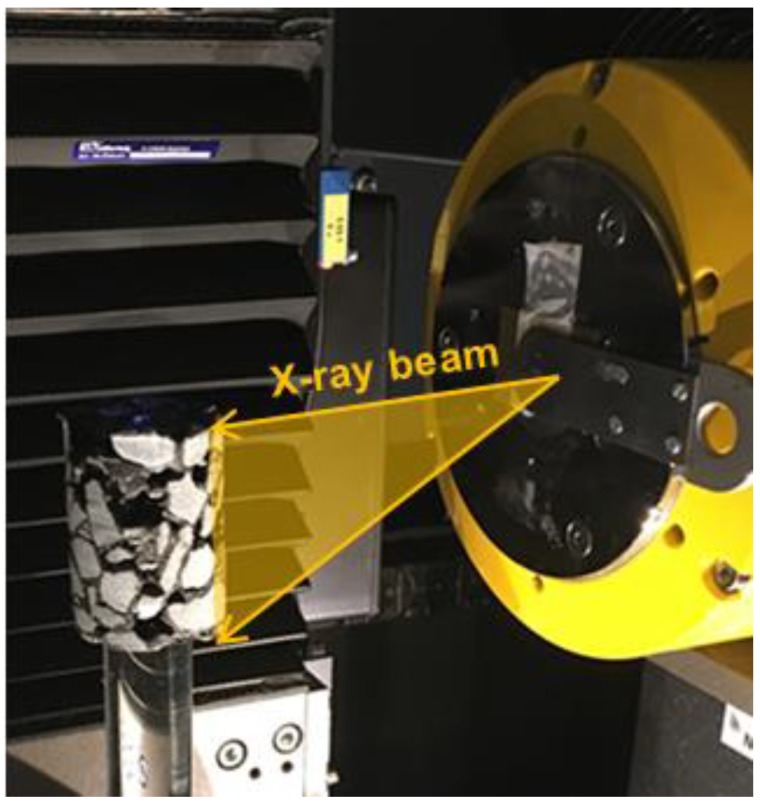
XCT scanning system for porous asphalt specimen.

The aim of using X-ray computed tomography (XCT) was to visualize the calcium alginate capsules within the porous asphalt mix. In this way, whether the capsules could survive the real asphalt production process and how they were distributed could be investigated. In this paper, the XCT was performed using a Phoenix Nanotom CT scanner (Baker Hughes, Wunstorf, Germany) (Figure 4). To fit the lateral dimension of the small porous asphalt cylinder, the resolution was set as 20 μm between each computed voxels.

#### 2.2.2. ITSM Tests

ITSM test, according to the standard EN 12697-26:2012 [23], was employed to investigate the effect of the alginate capsules encapsulating rejuvenator on the stiffness modulus of the Porous Asphalt mix. The porous asphalt cylinder specimens used in the ITSM tests had a diameter of 100 ± 2 mm and height of 50 ± 1 mm. A Universal Testing Machine (Industrial process controls LTD, Melbourne, Australia) with a maximum load of 5 kN (UTM 5) with a temperature chamber was employed in the ITSM tests. The ITSM tests were performed at 20 °C at four different frequencies (8 Hz, 4 Hz, 2 Hz and 1 Hz). The Poisson’s ratio of 0.22 was assumed for this porous asphalt concrete [21].

#### 2.2.3. SCB Tests

**Figure 5:**
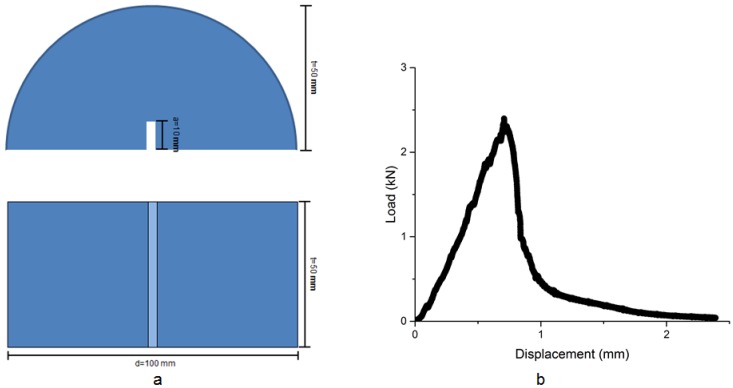
(**a**) Geometry information of the semi-circular porous asphalt specimens and (**b**) load vs displacement curve from SCB tests.

The SCB tests were performed according to EN 12697-44:2010 [24]. Figure 5a shows the schematic diagram of the semicircular test samples. The SCB samples had a diameter of 100 ± 2 mm, thickness of 50 ± 1 mm and radius of 50 ± 1 mm. A notch was placed in the middle of each sample, with a notch length of 10 ± 0.2 mm and width of 3 ± 0.1 mm. The loading speed was set as 5 mm/min. In order to achieve a brittle fracture from the notch throughout the test specimen, the SCB tests were performed in a temperature controlled chamber (Industrial process controls LTD, Melbourne, Australia) at 0 °C. A load vs displacement curve acquired from SCB tests is presented in Figure 5b. It indicated that the SCB tests were able to generate brittle fracture in the porous asphalt specimens.

**Figure 6:**
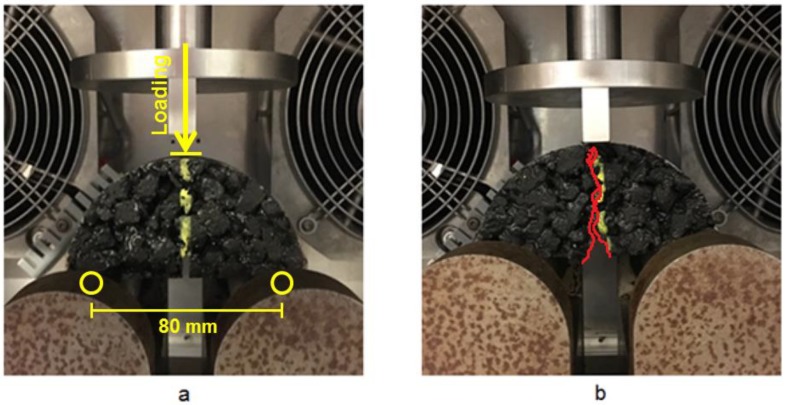
(**a**) SCB tests setup and (**b**) Fractured specimen after test.

A UTM 15 testing system with temperature chamber was employed to perform the SCB tests (Figure 6). The support span for the SCB tests was set as 80 mm, which was 80% of the diameter of the specimen (Figure 6a). Figure 6b shows a fractured specimen after the SCB test, which indicates that the SCB tests allow crack initiate from the notch, propagate throughout the depth and finally lead to fracture.

In order to evaluate the fracture resistance of the specimens, the peak load, fracture toughness and fracture energy were also calculated from the SCB tests results.

The fracture toughness (*K_Ic_*) and fracture energy (*G_Ic_*) were calculated following the NCHRP09-46 (for straight line notch), and using Equations (1) and (4), respectively:

(1) $$K_{Ic}=Y_{Ic\left(0.8\right)}\sigma_{o}\sqrt{\pi a}$$

where:

*K_Ic_* = Fracture Toughness (Pa·m^1/2^)

(2) $$\sigma_{o}\;=\;\mathrm{Stress}\;(N/m^{2}),\;\frac{P}{2rt}$$

*P* = Applied load (N)
*r* = Specimen radius (m)
*t* = Specimen thickness (m)
*Y_Ic_* = Normalised Stress intensity factor,

(3) $$Y_{Ic\left(0.8\right)}=4.782+1.219\left(\begin{array}{c} a\\ r \end{array}\right)+0.63e^{\left(7.045\left(\begin{array}{c} a\\ r \end{array}\right)\right)}$$

(4) $$G_{Ic}=\;\frac{W_{f}}{A_{lig}}$$

where:

*G_Ic_* = Fracture Energy (J/m^2^),
*W_f_* = Work of the fracture–area under the load displacement curve (J),

(5) $$W_{f}=\;\int Pdu$$

*P* = Applied load (N),
*u* = load line displacement (m),

A*_lig_* = Ligament area (m^2^), A*_lig_* = (r − a) × t (6)

r = Specimen radius (m),
t = Specimen thickness (m),
a = Notch length (m).

#### 2.2.4. Healing Efficiency Evaluation

In this research, evaluation of the healing efficiency was conducted with a testing and healing programme based on SCB tests. To evaluate the healing efficiency of a SCB specimen:

1. First, the initial peak load of the specimen were measured by the first SCB test;
2. Second, the fractured specimen was spliced to close the fracture face and conditioned at 23 °C for 20 h on a plain surface. In order to create a constant confinement to ensure the close of cracked surfaces, the specimens were carefully wrapped with tape during the healing process;
3. Subsequently, the second SCB test was performed to acquire the regained peak load of the specimen after healing. Afterwards, step 2 was repeated to perform another healing cycle following by the third SCB test.

Healing efficiency of the specimen was determined using healing index (HI), which was calculated with the peak load measured from three SCB tests, using equation (7):

(7) $$\mathrm{HI}=\frac{C_{x}}{C_{1}}$$

where:

HI = the healing index (%),
C_1_ = original peak load of the sample;
C_x_ = fracture property after x cycles of healing.

## 3. Results and Discussion

### 3.1. XCT

**Figure 7:**
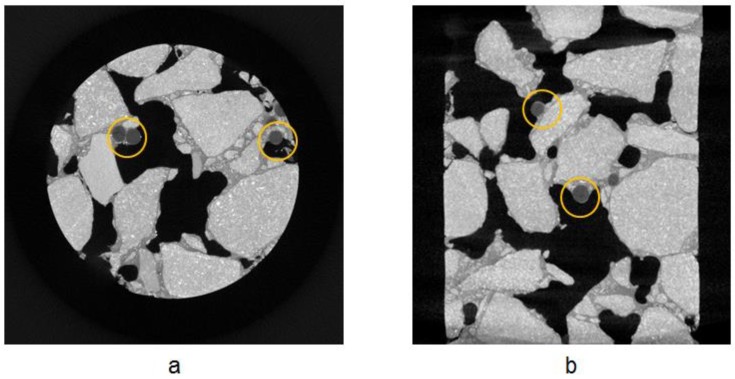
X-ray tomography images: (**a**) top view of the porous asphalt cylinder and (**b**) front view of porous asphalt cylinder.

Figure 7 shows the XCT scanning images of the porous asphalt sample. The XCT images clearly illustrate the porous structure and the material distribution of the porous asphalt mix. In both images, the calcium alginate capsules, which recognized as black round spots, are uniformly distributed in the porous structure together with asphalt mastic. The capsules within the structure preserve perfect round shape, which indicate that capsules are not damaged from the porous asphalt manufacture process. Meanwhile, the uniform distribution of capsules provides a higher chance to bring the capsules to the potential damaging sites, thus more comprehensive healing.

### 3.2. Indirect Tensile Stiffness Modulus of Porous Asphalt Concrete

**Figure 8:**
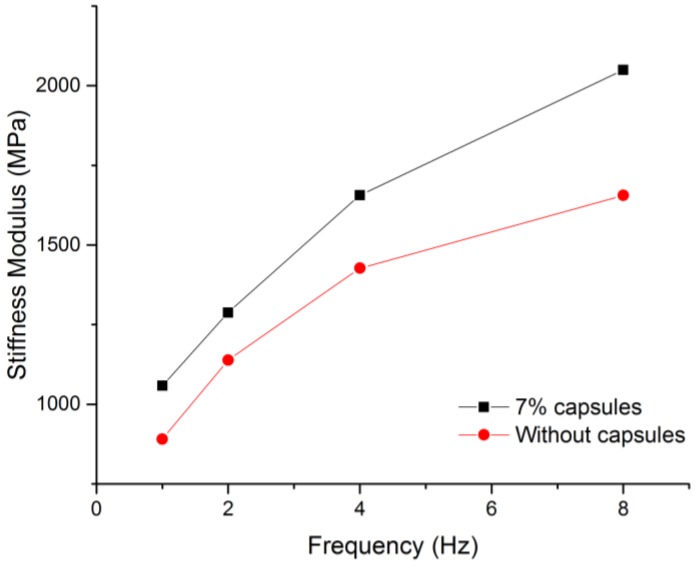
The stiffness modulus results at 20 °C.

Figure 8 shows the stiffness modulus results at 20 °C. The results show that with calcium alginate capsules, the porous asphalt specimens have higher stiffness modulus than reference specimens in all loading frequency. It indicates that adding the alginate capsules encapsulating the rejuvenator in porous asphalt mix contributes to a higher stiffness, which means that the capsules have a reinforcing effect on the porous asphalt concrete, similarly as for the asphalt mastic mix [9].

### 3.3. Fracture Face of the Porous Asphalt Specimens after the SCB Test

**Figure 9:**
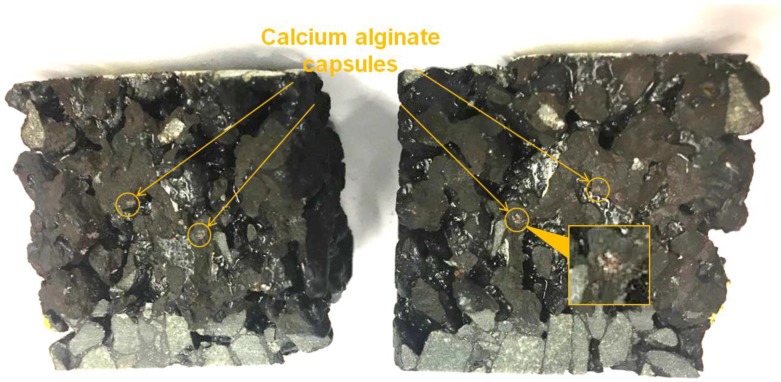
Fracture surface of a SCB specimen with 7% capsules.

The fracture surface of a SCB specimen with capsules is shown in Figure 9. As shown in Figure 9, broken capsules can be found on both sides of the fractured beams, which indicates that the capsules are able to break upon propagation of cracks. On the other hand, presences of these capsules demonstrate that the calcium alginate capsules have not been crushed by mixing or compaction in this research, which indicates a huge potential for the application in field construction.

**Figure 10:**
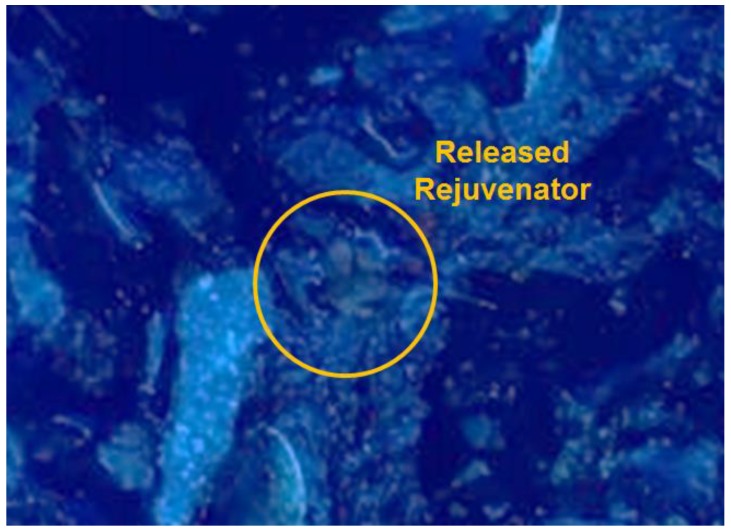
Released rejuvenator on fracture face.

In order to track the releasing of rejuvenator from the capsules which were broken in SCB tests, as shown in Figure 10, the sample on the right side of Figure 9 was exposed to UV light. Since the rejuvenator content of a single capsule is limited, there is no large area distribution of rejuvenator. However, following the locations of broken capsules in Figure 9, brown area can be found which refers to released rejuvenator under UV light.

### 3.4. Fracture Properties of the Porous Asphalt Concrete with Capsules

#### 3.4.1. Peak Load

**Figure 11:**
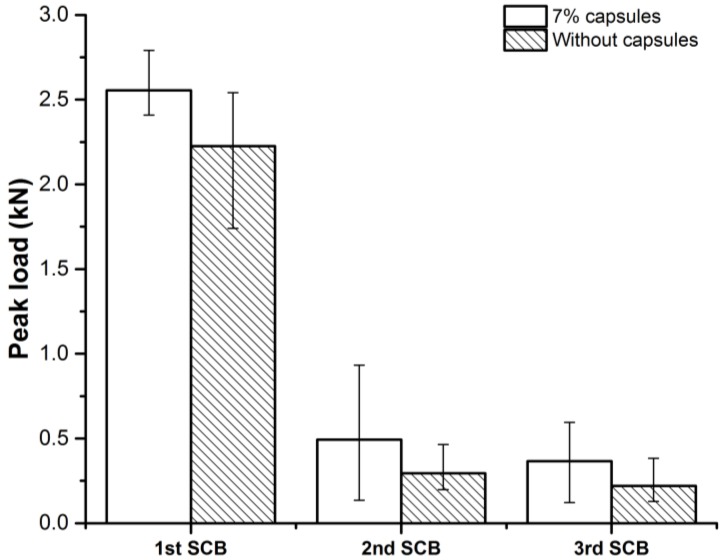
Peak load of SCB specimens.

Figure 11 shows the peak load results from SCB tests. Generally, the porous asphalt specimens with capsules have relatively higher peak load than the reference specimens and the increase is more significant in the 2nd and the 3rd SCB tests. Which might be because of the reinforcing effect from capsules that contribute to the facture resistance of the porous asphalt. The initial peak loads of both types of mix are much higher than the peak load from second and third SCB tests. Which means the 20 h healing period are not able to perform significant healing effect. It might because of the initial SCB test breaks the original arrangement of the aggregates of porous asphalt, and it is very hard to regain the interlocking effect between aggregates under room temperature and without any compaction. The peak loads from the second time SCB tests for the same specimen are very close to the peak loads from the third time SCB tests. It indicates that except defects from first SCB tests, the second time SCB tests did not seriously defect the porous asphalt specimens, as such the healing effect plays a more important role. That is also the reason that specimens with capsules show much higher fracture resistance in the second and third time SCB tests.

#### 3.4.2. Fracture Toughness

**Figure 12:**
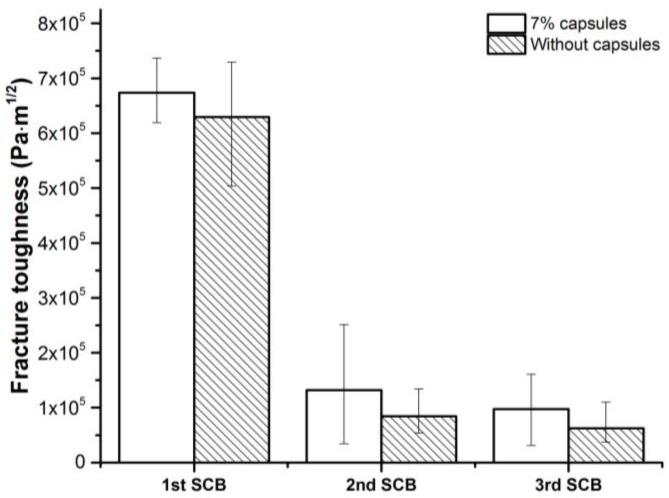
Fracture toughness of SCB specimens.

The fracture toughness of porous asphalt specimens, which directly represents the ability for the fracture resistance, are presented in Figure 12. As shown in Figure 12, due to the fracture damage, the initial toughness cannot be significantly healed in the following healing cycles, which is similar to the trend in Figure 11. However, SCB specimens with calcium alginate capsules show slightly higher toughness than reference specimens in all testing cycles. In the first time SCB tests, specimens with or without capsules does not show significant difference in toughness. While in the second time SCB tests, the specimens with capsules have an average toughness of 1.3 × 105 Pa·m^1/2^, but specimens without capsules have only 8.4 × 104 Pa·m^1/2^. Damages from the first time SCB tests have significant effect on the toughness of the SCB specimens and most of the toughness loss cannot be recovered. With calcium alginate capsules, the SCB specimens are able to achieve more healing in toughness.

#### 3.4.3. Fracture Energy

**Figure 13:**
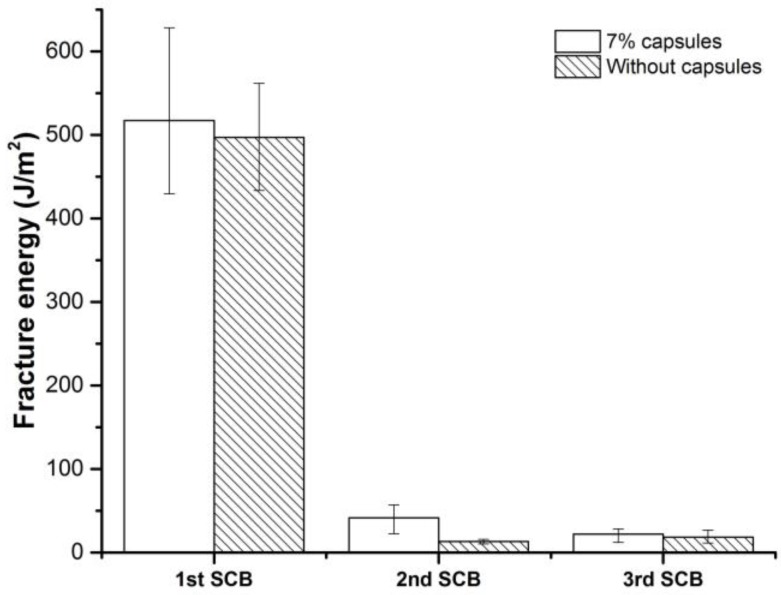
Fracture energy of SCB specimens.

Figure 13 presents the fracture energy results from SCB tests. The fracture energy calculation considers the area beneath the load-displacement curve, which is indicative of fracture resistance in the whole testing process that from the initiation of crack to the failure of sample. Similar to peak load and toughness, the first time SCB test results are very close, Nevertheless, SCB specimens with capsules consumes significantly higher energy than reference specimens. It indicates that the encapsulated rejuvenator released upon fracture, wet the fracture face and generate more bonds than reference specimens. Hence the SCB specimens with capsules accumulate more energy during the rest period and show much more healing than reference samples.

#### 3.4.4. Healing Efficiency

**Figure 14:**
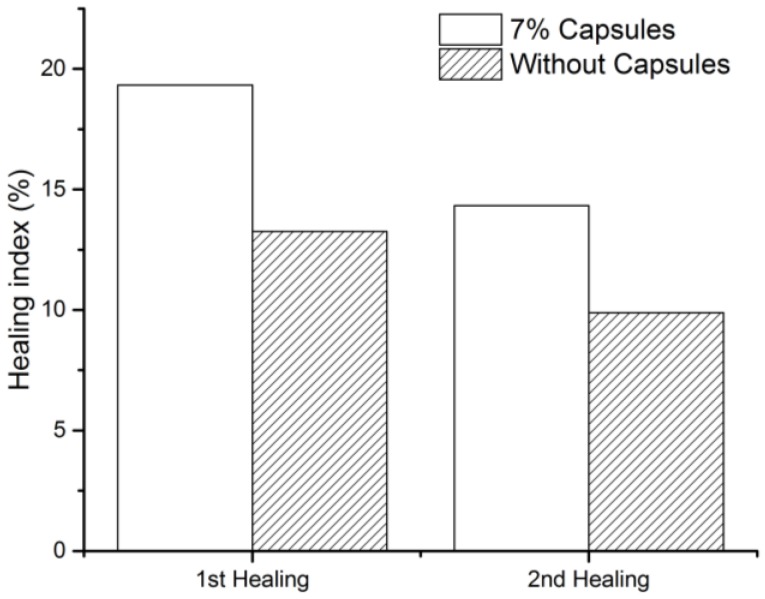
Healing index of SCB specimens.

In this research, the healing efficiency was evaluated by the peak load healing ratio during rest periods. The healing results are presented in Figure 14. The results show that the SCB specimens with capsules are able to restore 19.3% of the initial peak force, which is 6% higher than the specimens without capsules. In the third time SCB tests, SCB specimens with capsules can still achieve a healing ratio of 14.3%. Without capsules, the healing effect is only 9.9%. Figure 14 also illustrates that even with calcium alginate capsules, the healing effect on such a serious fracture is very limited. Although the application of calcium alginate capsules largely improved the healing capacity, the limitation of these capsules as well as other rejuvenation methods lies on the damage level. The calcium alginate capsules are more capable of micro-crack healing, aimed to close crack at early stage thus preventing serious defect in asphalt pavement. In this way, the calcium alginate capsules possess a healing potential in the application in construction field.

This research demonstrates that addition of calcium alginate capsules with encapsulated rejuvenator increases the healing efficiency of porous asphalt mix. However, this healing effect will decrease if a damage is beyond the healing capacity.

## 4. Conclusions

This study explores the potential use of calcium alginate in porous asphalt and following conclusions are drawn:

- XCT is an effective method to analyze the structure and material distribution in porous asphalt mix. In XCT images, the presence of capsules in the porous asphalt mix demonstrate that calcium alginate capsules are able to survive asphalt manufacture process. The uniform distribution of capsules indicates a more comprehensive healing potential in porous asphalt;
- From the ITSM tests results, application of these calcium alginate capsules shows reinforcing effect by improving the stiffness modulus of the porous asphalt mix;
- After SCB tests, the presence of capsules on the fracture surfaces indicates that calcium alginate capsules are able to fracture upon the propagation of cracks. By this means, the encapsulated rejuvenator are able to release and heal the damage site;
- In the SCB testing and healing programme, samples with capsules are able to achieve a healing index that is 6% higher than the reference samples, which means addition of calcium alginate capsules improves the healing capacity of asphalt during the porous asphalt fracture and healing cycles;
- However, samples with capsules could only achieve a maximum healing index of 19% in the healing cycles, which indicates that if the damage beyond the healing capacity, the calcium alginate capsules are not able to recover all the loosing properties especially interlocking effect between aggregates;
- The fluorescent component of rejuvenator allows tracking of the transportation of rejuvenator, which in turn could evaluate the healing effect.

Finally, this research initially explores the potential use of calcium alginate capsules in porous asphalt, the authors will continue the research on investigation of capsules on fatigue properties of PA and optimization of the calcium alginate capsules healing system. In comparison to the induction healing method, calcium alginate capsules show limited healing capacity of a PA. Thus, the future research will be focused on the development of a combined healing system which calcium alginate capsules are incorporated in induction healing to achieve an optimum healing which combines both effective crack healing and aged binder rejuvenation.

## Figures and Tables

**Table 1:** Mix composition of Porous Asphalt Concrete.

Mix Constituent	% Content in Mix	
	7% Capsules	Without Capsules
16 mm	8.5	8.5
11.2 mm	66.1	66.1
8 mm	8.5	8.5
5.6 mm	1.9	1.9
2 mm	6.9	6.9
500 μm	2.2	2.2
180 μm	0.7	0.7
125 μm	0.7	0.7
63 μm	4.5	4.5
Bitumen (70/100)	4.092	4.4
Capsules	0.363	0
